# Self-assessed and Clinical Halitosis among Patients in the UAE: Agreement and Associated Risk Factors

**DOI:** 10.3290/j.ohpd.c_2366

**Published:** 2025-12-02

**Authors:** Sabha Nasir Al-Qaydi, Roba Saqan, Najlaa Al-Bluwi, Xavier Struillou, Zahi Badran, Betul Rahman

**Affiliations:** a Sabha Nasir Al-Qaydi Periodontics Specialist, Emirates Health Services, Sharjah, UAE. Devised the project, contributed to conception, performed the clinical examinations, data collection and tests, drafted the manuscript, interpreted the results.; b Roba Saqan Research Assistant, Research Institute for Medical and Health Sciences, University of Sharjah, Sharjah, UAE. data analysis, drafted the manuscript, interpreted the results.; c Najlaa Al-Bluwi Graduate Research Assistant, Research Institute for Medical and Health Sciences, University of Sharjah, Sharjah, UAE. data analysis, drafted the manuscript, interpreted the results.; d Xavier Struillou Associate Professor, Inserm, UMR 1229, RMeS, Regenerative Medicine and Skeleton, University of Nantes, ONIRIS, Nantes, France, Department of Periodontology, Faculty of Dental Surgery, University of Nantes, Nantes, France. Critically revised and formated the manuscript.; e Zahi Badran Professor, Department of Restorative Dentistry, College of Dental Medicine, University of Sharjah, Sharjah, UAE. Critically revised and formated the manuscript.; f Betul Rahman Associate Professor, Department of Restorative Dentistry, College of Dental Medicine, University of Sharjah, Sharjah, UAE. Study idea and concept, drafted the manuscript, interpreted the results.

**Keywords:** halitosis, Oral Chroma, oral hygiene, public health, self-assessment, volatile sulfur compounds.

## Abstract

**Purpose:**

To evaluate the relationship between self-assessed and clinically measured halitosis, identify associated risk factors, and assess the diagnostic accuracy of a simple self-assessment method.

**Materials and Methods:**

A cross-sectional study was conducted among 102 adults in the UAE. Self-perceived halitosis was assessed via questionnaire and the cupping-hands technique (0–5 scale). Clinical halitosis was determined using Oral Chroma (Nissha FIS; Osaka, Japan) to measure volatile sulfur compounds (VSCs). Oral examinations recorded oral hygiene status, tongue coating, periodontal health, caries, and restoration quality. Associations were analyzed using bivariate and multivariate regression. The diagnostic accuracy of self-assessment was evaluated against Oral Chroma findings.

**Results:**

Self-reported halitosis prevalence increased from 26% (questionnaire) to 57.8% (cupping-hands), while clinical halitosis was detected in 63.7% of participants. Self-assessment showed moderate agreement with clinical diagnosis (κ = 0.426) and a sensitivity and specificity of 73.9% and 70.3%, respectively. Methyl mercaptan had the strongest association with perceived odor (AUC = 0.782). Both self-assessed and clinical halitosis were statistically significantly associated with tongue coating, poor oral hygiene, gingival bleeding, periodontal pockets, caries, imperfect restorations, and BANA positivity (all p < 0.05). Multivariate analysis identified smoking, prosthesis use, infrequent dental visits, and inadequate use of dental floss and mouthwash as independent predictors.

**Conclusion:**

Halitosis was highly prevalent and strongly linked to modifiable oral hygiene and behavioral factors. The cupping-hands method correlated with VSC measurements and may be a useful screening tool. Public health strategies and clinical practice should emphasize tongue cleaning, periodontal care, and regular dental visits to reduce oral malodor.

Halitosis is an unpleasant odor released from breath regardless of the origin, whether it originates from oral or systemic sources.^7,42^ It is considered a frequent chief complaint of patients.^3^ Halitosis affects all age groups and may be a temporary or chronic problem, depending on the underlying causes.^31^ When oral malodor is severe, it may negatively affect self-confidence and social interactions of those suffering from it.^30^ This may result in psychological changes leading to social and personal isolation.^15^

The source of malodor in 90% of patients is related to the oral cavity. However, approximately 9% of halitosis cases may be related to non-oral causes such as respiratory, digestive, or urinary system conditions. Furthermore, 1% of halitosis cases may be related to diet or medication.^5^ Halitosis may manifest from tongue coating, periodontal diseases, substantial carious cavities, infected extraction wounds, food impaction within large interdental areas, or dental crowding resulting in food entrapment. Furthermore, acrylic dentures, if not regularly cleaned and worn at night, may result in a typical odor related to candidiasis. Pericoronitis and mucosal ulcers are other causes of halitosis.^8^

Volatile sulfur compounds (VSCs) are the chief compounds leading to halitosis. These are primarily hydrogen sulfide (H₂S), methyl mercaptan (CH₃SH), and dimethyl sulfide ((CH₃)₂S).^41^ Porphyromonas gingivalis, Treponema denticola, and Tannerella forsythia are examples of Gram-negative anaerobic bacteria that produce these malodor compounds, and their presence can be detected using the N-benzoyl-DL-arginine-2-napthilamide (BANA) test.^32^

Among the intra-oral causes, periodontal disease is a major contributor to halitosis. Periodontitis is a highly prevalent, multifactorial, chronic inflammatory condition initiated by a dysbiotic biofilm and sustained by the host’s inflammatory response, resulting in progressive destruction of the periodontal apparatus.^8,44^ It is now widely recognized not only as a primary cause of tooth loss but also as a systemic condition with significant links to cardiovascular, metabolic, respiratory, and reproductive health.^19, 44^ In the context of halitosis, inflamed periodontal pockets create anaerobic environments favoring the proliferation of proteolytic bacteria capable of producing high levels of VSCs, particularly methyl mercaptan, which is strongly associated with tissue permeability and disease progression.^14,15^

A recent consensus from the European Federation of Periodontology underscores the importance of recognizing oral malodor as a clinically relevant manifestation of periodontal disease and advocates for integrated diagnostic frameworks that capture both local and systemic health impacts.^37^Advances in periodontal diagnostics—including the analysis of saliva and gingival crevicular fluid—offer promising non-invasive approaches for monitoring inflammatory status and microbial composition in both periodontitis and halitosis.^13,23^ Saliva in particular has emerged as a practical medium for detecting periodontal pathogens and inflammatory mediators, enabling the simultaneous evaluation of periodontal health and halitosis biomarkers.^13^

Intra-oral halitosis can be measured clinically by either the organoleptic test or with certain devices. Even though the organoleptic test has been considered the gold standard for oral malodor assessment^32^—since it uses a human nose to smell the odor from the mouth and rank its intensity—its use in clinical practice has been questioned since the COVID-19 pandemic. On the other hand, gas chromatography devices have been used to measure the intensity of oral malodor based on levels of VSCs within the oral cavity.^32,41^

Previously, it was assumed that people could not detect their own bad breath because they became desensitized to it.^16^ Nevertheless, this does not prevent a person with possible oral malodor from detecting his or her own breath by a variety of methods, such as cupping hands over their mouth and nose, licking the hand, smelling dental floss after flossing, or rubbing fingers across the gingivae.^25^

Several studies have investigated halitosis prevalence and its associations with oral and general health worldwide. However, data remain scarce in the United Arab Emirates (UAE), particularly regarding the agreement between self-perceived halitosis and clinically measured malodor. Given the well-established role of periodontal disease in halitosis, this gap warrants exploration in local populations.

Therefore, the aim of present study was to investigate the agreement between self-assessed halitosis and clinically measured malodor (via VSC detection) among study participants in the UAE. The specific objectives were: 1. to evaluate the association of self-assessed and clinical halitosis with oral health status, oral health–related behavior, and sociodemographic factors; 2. to identify the sulfur compound most strongly contributing to an individual’s perception of halitosis; and 3. to investigate the association of halitosis with the presence of periodontal pathogens using BANA testing.

## MATERIAL AND METHODS

### Ethics Approval and Consent to Participate

The University of Sharjah’s Research Ethics Committee approved the study (REC-20-05-12-02-S). Information about the study was provided to the participants verbally and in writing. All participants signed the informed consent. The authors confirmed that all methods were carried out in accordance with relevant guidelines and regulations.

### Study Design and Participants

A cross-sectional study was conducted on 102 volunteers (39 males, 63 females) recruited by convenience sampling from patients, staff, and students at the University of Sharjah Dental Hospital. Participants provided informed consent and met the following criteria: aged 18–60 years and having ≥20 teeth. Pregnant women and individuals who had taken antibiotics within the last three months were excluded.

Sample size was calculated using MedCalc Statistical Software version 20 (MedCalc Software; Ostend, Belgium). Assuming a halitosis prevalence of 31% (case:control ratio 0.31:0.69), a sample size was powered to detect whether the diagnostic test could discriminate between cases and controls with an AUC of 0.70, an alpha level of 0.05 and 90% power, and accounting for 10% non-response, the calculated sample size was 104 participants. Variance of the AUC was approximated using the example of DeLong et al.^12^ An AUC of 0.70 was chosen as the target effect size, representing good discriminatory ability compared with random performance (AUC = 0.50).^28^

A questionnaire was completed by each participant who gave their consent to participate, followed by a clinical examination performed by a single dentist. Participants were informed that they could withdraw from the study at any time.

### Questionnaire

A structured questionnaire, developed by merging two previously tested and validated questionnaires,^2,4^ was administered chairside by the investigator. The questionnaire contained closed-ended questions organized into two sections: 1. socio-demographic characteristics: age, gender, educational level, and marital status; 2. medical and oral health history: medical history, oral hygiene practices, time since last dental visit, consumption of alcoholic beverages, intake of odorous food in the last 24 hours, and smoking status (including whether the participant had smoked within the last 12 hours). Additional items included a dichotomous (yes/no) question asking participants whether they believed they had bad breath, questions prior to treatment for oral malodor, and questions addressing the perceived impact of halitosis on workplace performance or social life. Individuals who answered “yes” (self-perceived halitosis) as well as those who answered “no” were included in the study.

### Self-Assessment of Halitosis (Cupping Hands Method)

Participants were first instructed to smell their hands to ensure they were odor-free. They were then asked to close their mouths for three minutes, following the procedure described by Rosenberg et al,^35^ before exhaling into cupped hands covering both the mouth and nose and inhaling through the nose. They scored their own oral malodor on a 0–5 scale: 0 = no odor; 1 = questionable odor; 2 = slight but noticeable odor; 3 = moderate odor; 4 = strong odor; 5 = extremely foul odor.^31^ A score of ≥ 2 was considered a positive result.

### Clinical Examination

Following self-assessment, participants underwent a series of clinical examinations that included: Dental and periodontal evaluation, detection of periodontal pathogens on the tongue (BANA test), and quantitative measurement of volatile sulfur compounds (VSCs) using a portable sulfide monitor (Oral Chroma, CHM-2, Nissha FIS; Osaka, Japan).

All clinical examinations were performed by one of the authors (SN), a resident in the Master of Dental Surgery (MDS) program in periodontology. Intra-examiner reliability was assessed using the intraclass correlation coefficient (ICC) with a two-way mixed effects model, (absolute agreement, average measures). The results showed excellent reliability (ICC = 0.961,95% CI: 0.561–0.997, p < 0.008), indicating a high level of consistency in repeated measurements by the same examiner.

Simplified oral hygiene index (OHI-S):^18^ Each selected tooth surface was scored 0–3 for both debris and calculus, with 0 indicating none and 3 indicating the most. The debris and calculus scores were averaged, then summed to yield the final OHI-S score (0–6), interpreted as: 0–1.2 = good; 1.3–3.0 = fair; 3.1–6.0 = poor.

Grading of Tongue Coating: Tongue coating was graded using a modified scale:^21,24^ 0 = none; 1 = light (≈10% of the surface); 2 = moderate (10–50%); 3 = severe (>50%).

BANA Test: The BANA test (BANA Met LLC; Ann Arbor, MI, USA) was employed to detect periodontal pathogens (Porphyromonas gingivalis, Treponema denticola, Tannerella forsythia). A tongue coating sample was placed on a moistened reagent strip containing the BANA substrate. If bacterial peptidase enzymes were present, they hydrolyzed the peptide analog N-benzoyl-DL-arginine-β-naphthylamide, producing a blue color proportional to bacterial load. Results were scored thusly: 0 = negative (no blue color); 1 = weak positive (faint blue); 2 = positive (distinct blue).

### Other Oral Health Assessments

The following were recorded: number of teeth present, carious lesions, defective restorations, mucosal ulcerations, prosthetic appliances, pericoronitis, and dry mouth. Periodontal assessment included the number of sites with probing depth ≥ 4 mm (six sites per tooth) using a UNC-15 probe and bleeding on probing (BOP, %). Mouth dryness was evaluated using the tongue depressor test,^46^ where adhesion of a tongue depressor to the buccal mucosa indicated dryness.

Gas chromatography (Oral Chroma): Oral Chroma was used to measure VSCs with high accuracy. A 1-ml disposable syringe was inserted two-thirds of the way into the oral cavity, and participants closed their mouths for 30 s before sample collection. The air sample was injected into the device, and concentrations of hydrogen sulfide (H₂S), methyl mercaptan (CH₃SH), and dimethyl sulfide ((CH_3_)_2_S) were displayed in ppb. Measurements were repeated twice and averaged. Manufacturer thresholds for halitosis diagnosis were: H_2_S ≥ 112 ppb, CH_3_SH ≥ 26 ppb, and (CH_3_)_2_S ≥ 8 ppb. Participants exceeding at least one threshold were classified as having clinical halitosis.

### Statistical Analysis

Data were analyzed using SPSS version 27 (IBM; Armonk, NY, USA). Continuous data were reported as mean ± SD or median (interquartile range), and categorical data as frequencies and percentages. Associations between self-assessed halitosis, Oral Chroma results, and categorical variables (including BANA test) were tested using chi-squared or Fisher’s exact tests. The Mann-Whitney U-test was used to compare median oral health parameters between groups with and without halitosis. Intra-examiner reliability was assessed using the ICC. Agreement between self-assessed halitosis and VSC-based diagnosis was evaluated using Cohen’s kappa, with interpretation according to Cohen’s guidelines. Receiver operating characteristic (ROC) curve analysis was conducted to assess the relationship between VSC levels and self-perceived halitosis, identifying which compound most influenced self-perception.

Simple and multiple logistic regression analyses were performed to identify factors associated with self-perceived and clinically diagnosed halitosis. Multiple regression models were adjusted for age, sex, educational status, and any variable with p < 0.2 in the univariate analysis. Statistical significance was set at p < 0.05.

## RESULTS

Among the 102 study participants, females accounted for 61.8% (n = 63) and males for 38.2% (n = 39). The age range was 19–52 years, with a mean age of 29.14 ± 7.03 years. More than half of the participants (56.9%, n = 58) had a university education, while 43.1% (n = 44) had a high school education or less. Most participants were single (59.8%, n = 61), medically fit, and not on any medication.

When participants self-assessed their oral malodor using the cupping-hands technique, scores were distributed as follows: 22.5% (n = 23) reported no odor (score 0), 19.6% (n = 20) noted questionable odor (score 1), 30% (n = 31) detected slight odor (score 2), 21.6% (n = 22) reported moderate odor (score 3), and a few reported strong odor (score 4). Participants with a score of ≥ 2 were classified as having halitosis. Based on this threshold, 57.8% (n = 59) were identified with halitosis, while 42.2% (n = 43) had no halitosis. Using VSC detection with the Oral Chroma device, 63.7% of participants were found to have clinical halitosis (Table 1).

**Table 1:** Description of self-assessed halitosis and Oral Chroma halitosis

	No halitosis N (%)	Halitosis N (%)
Self-assessed halitosis	43 (42.2%)	59 (57.8%)
Oral Chroma halitosis	37 (36.3%)	65 (63.7%)


### Association with Sociodemographic and Oral Hygiene Factors

Table 2 shows the associations between self-assessed halitosis and sociodemographic variables, medical status, dental status, and oral hygiene practices. Chi-squared and Fisher’s exact tests revealed statistically significant associations between self-assessed halitosis and oral hygiene practices, including toothbrushing frequency, dental floss usage, regular mouthwash use, and daily tongue brushing (all p < 0.001).

**Table 2:** Characteristics of participants and their association with self-assessed halitosis categories (halitosis absent 43 [42.2%], halitosis present 59 [57.8%])

Sociodemographic and participant characteristics	Self-assessed halitosis	p-value
absent n= (43)	present n= (59)
Sociodemographic factors	Total N (%)	N (%)	N (%)
General health problems
Behavioral factors
Oral hygiene practices
Age	Mean ± SD	29.14±7.03	28.30 ± 7.37	29.75 ± 6.77	0.308
Gender	Male	39 (38.2%)	20 (51.3%)	19 (48.7%)	0.142
	Female	63 (61.8%)	23 (36.5%)	40 (63.5%)	
Marital status	Single	64 (62.7%)	30 (46.9%)	34 (53.1%)	0.210
	Married	38 (37.3%)	13 (34.2%)	25 (65.8%)	
Educational level	School	44 (43.1%)	20 (45.5%)	24 (54.5%)	0.557
	University or College	58 (56.9%)	23 (39.7%)	35 (60.3%)	
Diabetes	No	100 (98%)	43 (43%)	57 (57%)	0.507
	Yes	2 (2%)	0 (0%)	2 (100%)	
Gastrointestinal disease	No	94 (92.2%)	42 (44.7%)	52 (55.3%)	0.134
	Yes	8 (7.8%)	1 (12.5%)	7 (87.5%)	
Smoking status	Never smoked	93 (91.2%)	42 (45.2%)	51 (54.8%)	0.075
	Current smoker	9 (8.8%)	1 (11.1%)	8 (88.9%)	
Smoking in last 12 h	Yes	9 (8.8%)	1 (11.1%)	8 (88.9%)	
Gum chewing	No	83 (81.4%)	36 (43.4%)	47 (56.6%)	0.603
	Yes	19 (18.6%)	7 (36.8%)	12 (63.2%)	
Garlic, onion, or spicy food consumption in last 24 h	No	90 (88.2%)	42 (46.7%)	48 (53.3%)	0.012
	Yes	12 (11.8%)	1 (8.3%)	11 (91.7%)	
Toothbrushing frequency	Once a day or less	59 (57.8%)	15 (25.4%)	44 (74.6%)	<0.001
	Twice a day or more	43 (42.2%)	28 (65.1%)	15 (34.9%)	
Dental floss usage	Never	42 (41.2%)	9 (21.4%)	33 (78.6%)	<0.001
	Sometime or daily	60 (58.8%)	34 (56.7%	26 (43.3%)	
Toothpicks used regularly	No	78 (76.5%)	29 (37.2%)	49 (62.8%)	0.066
	Yes	24 (23.5%)	14 (58.3%)	10 (41.7%)	
Miswak usage	No	83 (81.4%)	35 (42.2%)	48 (57.8%)	0.996
	Yes	19 (18.6%)	8 (42.1%)	11 (57.9%)	
Mouthwash used regularly	No	65 (63.7%)	19 (29.2%)	46 (70.8%)	<0.001
	Yes	37 (36.3%)	24 (64.9%)	13 (35.1%)	
Daily tongue brushing or tongue cleaner use	No	51 (50%)	13 (25.5%)	38 (74.5%)	<0.001
	Yes	51 (50%)	30 (58.8%)	21 (41.2%)	
Time of last visit to dentist	<12 months	64 (62.7%)	27 (42.2%)	37 (57.8%)	0.994
	≥12 months	38 (37.3%)	16 (42.1%)	22 (57.9%)	


### Oral Health Status and Halitosis

Both self-assessed and clinical halitosis were statistically significantly associated with several oral health indicators: presence of a prosthesis (p = 0.007 and p = 0.004, respectively), tongue coating grade (p = 0.030 and p < 0.001), OHI-S (p = 0.007 and p < 0.001), gingival bleeding index (%) (p = 0.030 and p < 0.001), number of sites with probing depth ≥ 4 mm (p = 0.037 and p < 0.001), and number of carious teeth (p < 0.001 for both measures). Clinical halitosis, but not self-assessed halitosis, was also statistically significantly associated with the number of teeth with imperfect restorations (p < 0.001). The BANA test was statistically significantly associated with both self-assessed (p = 0.008) and clinical halitosis (p < 0.001).

### Agreement Between Self-Assessment and Clinical Diagnosis

Cohen’s kappa analysis indicated moderate agreement (κ = 0.426, p < 0.001) between self-assessed halitosis and Oral Chroma results (Table 3). The two methods agreed on 48 participants with halitosis and 26 without halitosis, suggesting comparable ability to correctly identify positive cases and reasonable precision for negative cases.

**Table 3:** Measurement of agreement (Kappa test) between the two methods (Oral Chroma evaluation of halitosis and self-assessed halitosis) (N=102)

	Oral Chroma halitosis N (%)	Total
absent		present
Self-assessed halitosis	Absent	26	70.30%	17	26.20%	43	42.20%
present	11	29.70%	48	73.80%	59	57.80%
Total	37	100%	65	100%	102	100%


### Diagnostic Accuracy of Self-Assessment

When compared with Oral Chroma results, self-assessment demonstrated a sensitivity of 73.85% (95% CI: 61.46–83.97) and specificity of 70.27% (95% CI: 53.02–84.13) (Table 4). The positive predictive value (PPV) was 81.36% (95% CI: 72.26–87.97), indicating that self-assessment correctly identified halitosis in over 80% of cases where it was reported. The negative predictive value (NPV) was 60.47% (95% CI: 49.14–70.77), indicating that self-assessment was less reliable in ruling out halitosis.

**Table 4:** Sensitivity and specificity of self-assessed halitosis compared to halitosis determined by chroma test (N=102)

	Value	95% CI
Sensitivity	73.85%	61.46% – 83.97%
Specificity	70.27%	53.02% – 84.13%
Positive predictive value	81.36%	72.26% – 87.97%
Negative predictive value	60.47%	49.14% – 70.77%


### Association with Sulfur Compound Levels

Self-assessed halitosis was statistically significantly associated with all three sulfur compounds used in clinical diagnosis—H_2_S, CH_3_SH, and (CH_3_)_2_S—with higher median (IQR) values in participants reporting halitosis compared to those without: H_2_S (72 [102] vs 133 [123], p < 0.001), CH_3_SH (20 [11] vs 38 [23], p < 0.001), and (CH_3_)_2_S (7 [5] vs 9 [48], p = 0.003) (Table 5).

**Table 5:** Comparison of self-assessed halitosis severity with clinical oral malodor levels measured by Oral Chroma

Self-assessed halitosis	Oral Chroma halitosis Median (IQR)
Clinical oral malodor (H2S) Cutoff: 112 ppb	Clinical oral malodor (CH3SH) Cutoff: 26 ppb	Clinical oral malodor ((CH3)2S) Cutoff: 8 ppb
No odor	98.0 (54)	20.0 (11)	7.0 (4)
Questionable odor	14.5 (63)	20.0 (36)	6.0 (8)
Slight / noticeable odor	117.0 (101)	36.0 (28)	7.0 (19)
Moderate odor	157.0 (96)	35.5 (35)	13.0 (45)
Strong odor	204.5 (120)	57.5 (39)	31.0 (180)
p-value	<0.001	<0.001	0.014


The Kruskal–Wallis test showed that median sulfur compound levels increased with higher self-reported odor severity. For H_2_S, median levels exceeded the clinical cutoff (112 ppb) beginning at the “slight but noticeable” category (117.0) and rose with increasing severity. CH_3_SH exceeded its cutoff (26 ppb) in the “moderate” and “strong” categories, while (CH_3_)_2_S exceeded its cutoff (8 ppb) in the “moderate” and “strong” categories. These findings support the validity of self-assessment in reflecting halitosis severity.

### ROC Analysis

**Fig 1:**
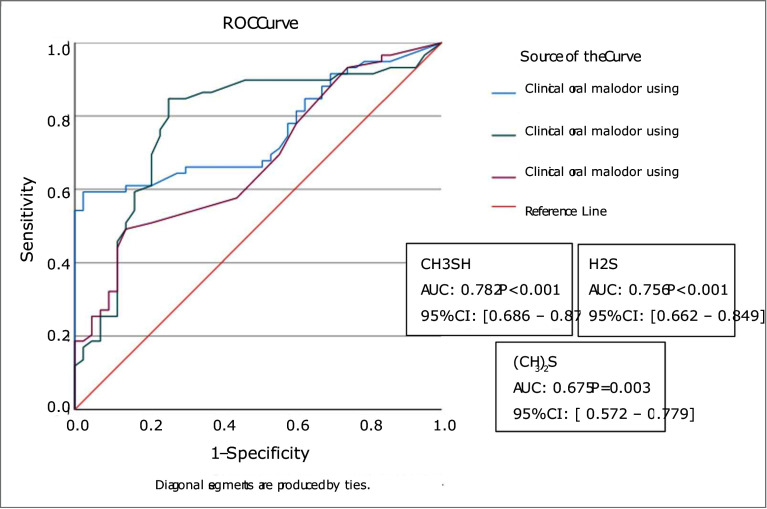
The association of self-reported halitosis and Oral Chroma volatile sulfur compounds (VSC).

ROC curve analysis demonstrated statistically significant associations between self-reported halitosis and all three sulfur compounds (Fig 1). Methyl mercaptan (CH₃SH) had the highest AUC (0.782, p < 0.001), suggesting it contributed most strongly to participants’ perception of oral malodor.

### Multiple Regression Analysis

Table 6 summarizes the multiple regression models, adjusted for age, sex, educational status, and variables with p < 0.2 in univariate analysis. Both self-assessed and clinical halitosis were statistically significantly associated with smoking status (OR = 83.02, 95% CI: 3.49–1975.00 and OR = 19.68, 95% CI: 1.34–289.33, respectively) and number of carious teeth (OR = 4.14, 95% CI: 1.8–9.52 and OR = 3.36, 95% CI: 1.55–7.28, respectively). Regular dental floss use was protective against both self-assessed (OR = 0.14, 95% CI: 0.04–0.55) and clinical halitosis (OR = 0.23, 95% CI: 0.08–0.69).

Self-assessed halitosis was additionally associated with higher education (OR = 4.84, 95% CI: 1.13–20.67), gum chewing (OR = 10.82, 95% CI: 1.56–75.06), less frequent dental visits (≥ 12 months) (OR = 4.57, 95% CI: 1.09–19.15), presence of a prosthesis (OR = 35.49, 95% CI: 2.46–512.90), number of teeth with imperfect restorations (OR = 4.20, 95% CI: 1.07–16.45), and lack of regular mouthwash use (OR = 0.06, 95% CI: 0.01–0.29). These factors were not statistically significantly associated with clinical halitosis. Participant age was positively associated with clinical halitosis (OR = 1.11, 95% CI: 1.02–1.21) but not with self-assessed halitosis.

The data produced during this research is available from the corresponding author on reasonable request.

## DISCUSSION

### Self-assessment of Halitosis

Self-assessment of oral malodor is inherently challenging, as individuals often cannot reliably detect their own breath odor.^32,35^ In this study, 26% of participants initially reported halitosis in the questionnaire, increasing to 58% after performing the “cupping hands” technique. This suggests that some participants were unaware of their malodor until prompted to self-assess, while others may have perceived halitosis without clinical evidence. These findings are consistent with previous reports that self-assessment can reveal unrecognized malodor but may also lead to false positives due to subjective bias and interference from hand odors.^4,8,20,29,32^

Our data showed a statistically significant correlation between self-assessed halitosis and volatile sulfur compound (VSC) levels measured by Oral Chroma. This aligns with studies by Romano et al^34^ and Pham et al,^32^ but differs from Bornstein et al,^7^ who found little or no association. ROC analysis further indicated that methyl mercaptan had the greatest impact on perceived odor among the detected sulfur compounds.

### Clinical Findings

Clinical halitosis was present in 63.7% of participants, a higher prevalence than reported in general population studies.^26^ Possible explanations include insufficient oral hygiene, tongue coating, periodontal pathogens (as indicated by BANA positivity), and deeper periodontal pockets. Tongue coating, gingival bleeding, and poor oral hygiene (high OHI-S scores) were statistically significantly associated with both self-perceived and clinically measured halitosis. These results support prior evidence that tongue dorsum biofilm and periodontal inflammation are major contributors to oral malodor.^2,5,10,27,38,43^

### Predictors of Self-Perceived Halitosis

Multivariate analysis identified nine predictors significantly associated with self-reported halitosis, including education level, smoking, prosthesis use, number of carious teeth, imperfect restorations, and infrequent dental visits. Lack of regular mouthwash and dental floss use were also associated with higher odds of self-reported halitosis. While some of these associations (e.g., OR > 30) were statistically significant, the wide confidence intervals indicate considerable uncertainty, and these estimates should be interpreted cautiously.

Interestingly, higher education was linked to increased self-reported halitosis but not to clinical halitosis, possibly reflecting greater awareness and self-monitoring among more educated individuals.^2,33^

### Comparison with Previous Research

The prevalence of self-reported halitosis in our study was higher than in many other studies.^1,31,38,40^ However, it was lower than in certain other populations.^7,21,32^ Cultural differences, variations in rating scales, and methodological differences—such as our use of a 0–5 cupping-hands scale vs visual analog scales—likely contribute to these discrepancies.^9,21^ Previous studies have also shown that females report halitosis more often than males, a trend observed in our data, possibly due to greater concern for oral hygiene and esthetics.^12,36^

### Limitations

The organoleptic test, considered a gold standard for halitosis detection, was not used due to its subjectivity, risk of nasal adaptation, and—importantly—its potential for disease transmission via exhaled air.^6,24^ This was a particular concern during the COVID-19 pandemic, when infection-control protocols discouraged close-contact odor assessment. Additionally, self-assessment methods may be influenced by skin odors from the hands and by psychological or cultural factors.^20^ The cross-sectional design and convenient sampling method also limits causal inference.

### Implications

Given the high prevalence of halitosis in this UAE sample, and the associations with modifiable oral hygiene behaviors, both dental and medical professionals should integrate halitosis prevention into patient education. Public health programs should address malodor within broader oral health promotion strategies, with emphasis on tongue cleaning, periodontal care, and routine dental visits.

## CONCLUSION

Self-assessment of halitosis using the cupping-hands technique showed a statistically significant correlation with VSC measurements from Oral Chroma, suggesting its potential as a simple awareness tool. However, subjective perception is not always reliable, as some individuals reported halitosis without clinical confirmation, while others were unaware of it until prompted to self-assess.

Clinically, halitosis was highly prevalent and strongly linked to tongue coating, periodontal inflammation, poor oral hygiene, and the presence of periodontal pathogens. Multiple behavioral and dental factors—including irregular dental visits, lack of tongue cleaning, and inadequate interdental hygiene—were predictive of self-reported malodor, highlighting modifiable risk factors.

From a public health perspective, halitosis warrants greater attention in both dental practice and community programs. Education on tongue cleaning, periodontal health, and routine dental care may reduce oral malodor prevalence. Future research should involve larger, representative populations and use both self-assessment and validated clinical measures to refine detection and management strategies.

## ACKNOWLEDGMENTS

The University of Sharjah supported this work. We acknowledge late Prof. Collin Alexander Murray for his valuable contribution to this research, and Dr. Fatima AlQetti for her assistance in recruiting participants for the study.

## REFERENCES
